# Impact of referral templates on the quality of referrals from primary to secondary care: a cluster randomised trial

**DOI:** 10.1186/s12913-015-1017-7

**Published:** 2015-08-29

**Authors:** Henrik Wåhlberg, Per Christian Valle, Siri Malm, Ann Ragnhild Broderstad

**Affiliations:** Department of Community Medicine, UiT The Arctic University of Norway, 9037 Tromsø, Norway; University Hospital of North Norway Harstad, St. Olavsgate 70, 9480 Harstad, Norway; Department of Clinical Medicine, UiT The Arctic University of Norway, 9037 Tromsø, Norway; Centre for Sami Health Research, UiT The Arctic University of Norway, 9037 Tromsø, Norway

## Abstract

**Background:**

The referral letter is an important document facilitating the transfer of care from a general practitioner (GP) to secondary care. Hospital doctors have often criticised the quality and content of referral letters, and the effectiveness of improvement efforts remains uncertain.

**Methods:**

A cluster randomised trial was conducted using referral templates for patients in four diagnostic groups: dyspepsia, suspected colorectal cancer, chest pain and chronic obstructive pulmonary disease. The GP surgery was the unit of randomisation. Of the 14 surgeries served by the University Hospital of North Norway Harstad, seven were randomised to the intervention group. Intervention GPs used referral templates soliciting core clinical information when initiating a new referral in one of the four clinical areas. Intermittent surgery visits by study personnel were also carried out. A total of 500 patients were included, with 281 in the intervention and 219 in the control arm. Referral quality scoring was performed by three blinded raters. Data were analysed using multi-level regression modelling. All analyses were conducted on intention-to-treat basis.

**Results:**

In the final multilevel model, referrals in the intervention group scored 18 % higher (95 % CI (11 %, 25 %), *p* < 0.001) on the referral quality score than the control group. The model also showed that board certified GPs and GPs in larger surgeries produced referrals of significantly higher quality.

**Conclusion:**

In this study, the dissemination of referral templates coupled with intermittent surgery visits produced higher quality referrals.

**Trial registration:**

This trial has been registered at ClinicalTrials.gov. The trial registration number is NCT01470963.

**Electronic supplementary material:**

The online version of this article (doi:10.1186/s12913-015-1017-7) contains supplementary material, which is available to authorized users.

## Background

A referral facilitates the transition of care from a general practitioner (GP) to secondary care to establish a diagnosis, to provide treatment including surgery, and to offer advice or reassurance. Hospital specialists frequently have complained about the perceived quality of referral letters. Several studies have highlighted the varying quality and content of referrals across a range of clinical specialities [1–12]. A recent Canadian survey of more than 3000 GPs and specialists found that, among the main problems specialists identified, 51 % of referral letters had an unclear reason for referral [13]. This variation in quality makes the evaluation and prioritisation of incoming referrals difficult, with one author stating that prioritisation cannot be performed based on referral letters alone [14].

A high quality referral process will generally involve referral letters containing all necessary information in a context of shared understanding between GPs, patients and hospital staff [15]. There have been previous definitions of what referral letters should contain [16]. In Norway, the Norwegian Centre for Informatics in Health and Social Care (KITH) has developed guidelines governing the content of electronic referral and discharge letters [17, 18]. However, these guidelines present headings and content categories, but do not specify the precise clinical information required for different clinical areas. A recent Norwegian study has highlighted that it is the lack of information in the referrals, rather than the structure and categories of the referral, that hospital doctors perceive as a barrier to high quality cooperative care [19]. To measure referral quality, it therefore seems necessary to focus more on measuring the informational quality of referrals than on measuring their structure. This conclusion is echoed by other publications in the field [8, 12, 20]. Several of these studies developed scoring systems collaboratively between hospital doctors and GPs [12, 20].

The introduction of electronic health records and communication has, to some extent, eliminated some of the structural problems with referral letters, but further work is needed to elucidate the relationship between the quality of clinical information in referrals and high quality health care processes. This is important as healthcare costs are rising globally [21] and services are being delivered within a framework of increasingly limited resources. In this context, it is imperative to improve patient prioritisation based on referrals in order to aid swift diagnosis in those with more serious disease and to provide evidence based high quality care to each individual patient. Tools to improve referral quality are paramount.

This paper reports the effects of a referral intervention on the quality of referrals in a cluster randomised trial. We hypothesized that the referral intervention would improve informational quality in the referrals. We assess whether other GP-related factors, including patient list size and years of experience, affect the quality of referrals written. This paper is part of a larger study assessing the effect of a referral intervention on the quality of health care delivered to individual patients. Information about further assessments within the referral project is available in the published methods paper [22].

## Methods

### Study setting

The Norwegian health care system is relatively uniform throughout the country. Each person has a regular GP who acts as a gatekeeper to secondary care [23]. GPs work either privately, with capitation payment and fee-for-service reimbursement, or as municipality employees. Specialist health care is delivered through governmentally owned regional health authorities, mainly in public hospitals. Some specialist outpatient care is purchased by the regional health authorities from private specialists, but access to this is very limited in the geographical area of the current study. Electronic health records are almost ubiquitous and referrals are sent according to a national standard that automatically includes demographic information including address, contact details and GP details [17].

### Study design

This study was designed as a cluster randomised trial with the general practitioner surgery as the clustering unit. All 14 community GP surgeries in the area served by the University Hospital of North Norway (UNN) Harstad were randomised to the intervention or control group. The cluster design was chosen to avoid contamination between GPs, which could have occurred if individual GPs at the same surgery were randomised to different groups.

The referring GP could not be blinded because the intervention was actively used by the GP. Patients, hospital doctors and outcome evaluators were blinded to the patient’s intervention status. However, in some cases the referral letter revealed the intervention status. Further information about study methods are available in detail in the methods paper [22].

### Intervention

The intervention consisted of the distribution of referral templates to the intervention surgeries. The templates were provided in paper and electronic forms. The templates were to be used when initiating a new referral to the medical outpatient clinic for patients within the four diagnostic areas specified below. These referral templates were developed based upon national and international literature [12, 24–31] and in collaboration with local specialists within each medical field. A clinical assessment process using specialists from other Northern Norwegian hospitals provided further insight into template contents. To ensure intervention implementation by keeping it as simple as possible, we reduced the number of items in the referral template to include only those that the specialists felt were imperative in a referral for that clinical area. The templates contained a heading soliciting further information in the referral about each item listed in the subsequent list of items. For example, the items in the referral template for patients with suspected colorectal cancer are shown in Table 1 (translated into English). The other templates are available in Additional file 1. The intervention offices were also provided with a separate electronic referral address at the hospital to enable study organisers to track the use of the intervention.

**Table 1:** Referral template for patients with suspected colorectal cancer

Item #	Item text
1	Change in bowel habit
2	Blood in stool
3	Weight loss
4	Family history of colorectal cancer
5	Previous medical history of bowel disease or results of previous bowel investigations
6	Results of digital rectal examination (DRE)
7	Iron deficiency anaemia
8	Clinical findings at abdominal examination
9	Result of faecal occult blood test (FOBT)
10	The general practitioners clinical suspicion

The templates were distributed by the corresponding author (HW) during educational and/or lunch meetings at the intervention surgeries. Prior to the distribution of templates, the project had been presented to the GPs at similar meetings. The intervention was in use for approximately 2 years, from September 2011 to November 2013.

Additional follow-up was provided in the form of lunchtime visits to the intervention surgeries approximately twice yearly and intermittent mail leaflets and reminders. The lunchtime visits were performed by HW and provided information about the progress of the study, reminders to use the intervention templates and answers to questions about the project. In addition, personal letters to participating doctors were sent when it was evident that the intervention had not been used in a received referral.

In the control group, normal referral practice continued. No information about the study was provided to the control surgeries.

Four separate diagnostic groups were selected; these represent both important clinical areas and a substantial amount of outpatient appointments.

- patients referred with dyspepsia
- patients referred with suspected colorectal cancer (CRC)
- patients referred with chest pain
- patients referred with confirmed or suspected chronic obstructive pulmonary disease (COPD)

At UNN Harstad in 2008 these diagnostic areas accounted for approximately 26 % of all patients in the medical outpatient clinics (own data), although separating new referrals from control patients in this material was not possible. In addition, patients in these clinical areas often represent a diagnostic challenge in primary care [32] and are well suited for simple referral templates.

### Participants

The 14 GP surgeries in the area primarily served by UNN Harstad were included in the randomisation process. In 2013, these surgeries had a total list size of 39,253 patients. Five surgeries were town-based and nine were rural. To ensure equal sociodemographic backgrounds between groups, the surgeries were randomised stratified by town or countryside location, although the location of the surgery itself was not expected to influence the main outcome variables. Two centres initially randomised to the intervention group declined to participate, one because of lack of interest and one because the GP was about to retire. Two further centres were therefore randomly selected. The final intervention group consisted of three urban and four rural surgeries, with two urban and five rural surgeries in the control group.

New patients referred to the UNN Harstad medical outpatient clinics in any of the four diagnostic groups received written information about the study and a participant consent form along with their appointment letter. Patients were orally reminded of the study by the hospital doctor at their hospital outpatient appointment and were given a new consent form if appropriate. Children (<18 years of age) and patients with reduced capacity to consent were excluded from the project. Further details about the GP surgeries and the recruitment process are published in the methods paper [22].

### Recruitment

**Fig. 1:**
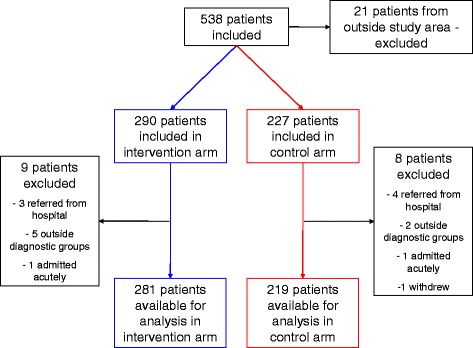
Inclusion process. Flow chart of the inclusion process

Recruitment ran for about 2.5 years, from September 2011 to February 2014, to ensure that patients referred during the project (the template was used until November 2014) had an outpatient appointment before inclusion closed. This timeframe was chosen because few patients at the hospital experience waiting times of >4 months from the time of their referral to the time of their hospital appointment. A total of 538 patients were included in the project. Thirty-eight patients were excluded because they did not fulfil the inclusion criteria, as depicted in Fig. 1. In total, 290 patients were included in the intervention arm and 227 patients in the control arm.

### Ethics

The study followed the directions in the Helsinki Declaration. Before recruitment started, the study was presented to the Regional Ethical Committee for Medical Research in North-Norway, who determined it not to be within the scope of the Health Research Act (REK NORD 2010/2259). The study was approved by the Data Protection Official for Research. The study is registered at ClinicalTrials.gov. The trial registration number is NCT01470963. All patients provided written informed consent.

### Sample size

For the overall study, sample size calculation was performed for the main outcome variable (a health care quality score), as shown in the methods paper [22]. No specific sample size calculation was performed for the referral quality outcome reported in this paper.

### Referral scoring

The referrals were rated according to a scoring system derived directly from the referral templates used. These templates specified the clinical information that specialists deemed most important in the referral, based on literature documented above. One point was awarded for the presence in the referral of each of the clinical details requested in the referral template. The maximum score possible for referrals for dyspepsia, suspected CRC, COPD and chest pain were 17, 10, 15 and 13 respectively. Other studies have also awarded points for the presence of core information including full contact details and legibility of referrals [33]. Because all but six referrals in this project were electronic, and such information is automatically included in the electronic referral, this information was not included in the scoring. The final referral score therefore represents how many of the information points in the template were actually articulated in the referral. Each score was then transformed to a percentage value.

Three raters scored the referrals. A sample of 100 out of the 500 referrals was scored independently by two raters. No referral was scored by all three raters, and all three rater pairs shared at least 25 referrals. The raters were blinded to the intervention status of the referring GP.

For further analysis, a small amount of data was imputed. For referrals initiated by interns, the list size was missing by default. Interns in Norway spend six months attached to a GP surgery where they do not have their own list of patients. Instead, they take part in the general workload of the surgery. For each referral initiated by an intern, the list size value in the dataset was set to the average list size value for the surgery the intern was attached to. This was believed to best represent the anticipated workload of each intern.

Speciality status was available for all referring GPs. Years of experience as a GP and in hospitals was available for 499 of 500 referrals, while data for the last case were imputed as mean values.

### Statistical analysis

Analyses were stratified by intervention group and control group. To ensure rater consistency an interrater reliability analysis using the weighted Kappa statistic was performed, as developed by Cohen [34]. Referral scores were divided into centiles, and a weighted Kappa analysis was performed using quadratic weights. Additional analysis was carried out for each rater pair separately and with the data divided into quintiles to ensure consistency of analysis.

We chose to use standard weights (quadratic) to improve interpretability, in concordance with discussion on the appropriate use of Kappa analysis [35]. In this way, the weighted Kappa coefficient approximates the intraclass correlation coefficient [36]. The weighted Kappa coefficient increases with increasing numbers of categories, especially when using quadratic weights [37]. However, this increase seems more pronounced in the range of two to five categories.

The cluster randomised design necessitates an analysis that is suitable for clustering. In this project, multi-level regression modelling was used. A stepwise approach was used to build the multi-level model. Likelihood ratio tests were used to evaluate whether random regression coefficients should be considered. Continuous variables were centred to facilitate interpretation. Because the intervention was randomised at the level of the GP surgery, no slope could be added for the intervention effect. To assess the addition of confounders to level one of the model, a change in the magnitude of the regression coefficient for the intervention effect of more than 10 % was considered indicative of a confound. In addition, the following variables were included based on prior subject knowledge: whether the referring GP was a specialist, the length of GP experience and GP list size. Effect modification was checked for relevant variables using *p* < 0.10 as the significance level. This level was chosen because the power to detect relevant interactions is often low, especially concerning cross-level interactions in multilevel studies [38]. Although increasing the type 1 error rate has been shown to be ineffective [39], it was judged to be better than missing important interactions. Analysis was done on an intention-to-treat basis, as recommended [40]. All referrals from intervention surgeries were therefore analysed as if they had used the referral intervention, even though it may have been evident that the intervention was not used. Stata version 13 (StataCorp. 2013. Stata Statistical Software: Release 13. College Station, TX: StataCorp LP) was used for all analysis.

## Results

### Baseline characteristics

Table 2 presents baseline characteristics of the study population. Patients ranged from 17 to 90 years of age. In both the intervention and control groups there were more women (59 and 58 %) than men (41 and 42 %). The majority of the referrals were in the dyspepsia group. Baseline characteristics for GP surgeries and referrals are available in Table 3. The groups appear similar, except that more referrals were initiated by male GPs in the control group than in the intervention group, which is probably caused by the slightly higher number of male GPs in the control group. Further, significantly more of the referrals in the intervention group than in the control group were made by GP specialists, thereby necessitating this as a covariate in the regression model. There were 37 referrals from interns, accounting for 7.4 % of the total number of referrals. A total of 139 of the 281 (49.5 %) intervention group referrals were sent to the designated electronic referral address created for the project; the rest were sent to the standard hospital electronic address.

**Table 2:** Selected patient baseline characteristics by intervention status

	Intervention group	Control group	*p*-value
Patient demographics^1^			
Female/male, n (%)	166 (59.07)/115 (40.93)	127 (57.99)/92 (42.01)	*p* = 0.807
Age, years	59.21 ± 13.64	57.08 ± 15.26	*p* = 0.101
Urban/rural, n (%)	169 (60.14)/112 (39.86)	121 (55.25)/98 (44.75)	*p* = 0.272
Clinical group, n (%)			
- dyspepsia	144 (51.25)	120 (54.79)	
- suspected colonic malignancy	87 (30.96)	68 (31.05)	
- chest pain	46 (16.37)	27 (12.33)	
- chronic obstructive pulmonary disease	4 (1.42)	4 (1.83)	
Hospital appointment with senior house officer/specialist, n (%)	130 (46.26)/151 (53.74)	96 (43.84)/123 (56.16)	*p* = 0.588

^1^Data are presented as mean ± SD or number (%)

**Table 3:** Selected general practitioner (GP) baseline characteristics by intervention status^1^

	Intervention group	Control group	*p*-value
GP surgery variables^2^			
List size	830.79 ± 208.78	865.48 ± 100.69	*p* = 0.475
Female/male GP, n (%)	14 (58.33)/10 (41.67)	10 (43.48)/13 (56.52)	*p* = 0.308
Specialist yes/no, n (%)	18 (75)/6 (25)	11 (47.83)/12 (52.17)	*p* = 0.055
Years experience	16.02 ± 10.40	15.15 ± 11.15	*p* = 0.784
Years experience in hospital	2.81 ± 5.94	1.89 ± 3.06	*p* = 0.510
Number of GPs in surgery	4.33 ± 1.61	4.04 ± 1.58	*p* =0.536
- median	5	5	
- mode	5	5	
GP referral variables per referral in dataset^2^			
Female/male referring GP, n (%)	182 (64.77)/99 (35.23)	93 (42.47)/126 (57.53)	*p* < 0.00001^4^
Number of GPs in surgery	4.43 ± 1.46	4.01 ± 1.62	*p* =0.003^3^
Specialist yes/no n (%)	189 (67.26)/92 (32.74)	114 (52.05)/105(47.95)	*p* = 0.000556^4^
Years experience	16.21 ± 11.96	15.41 ± 11.70	*p* = 0.456
Years experience in hospital	1.54 ± 1.88	1.54 ± 2.46	*p* = 0.994
Other variables per referral in dataset^2^			
Electronic/paper referral n (%)	281 (100)/0 (0)	213 (97.26)/ 6 (2.74)	*p* = 0.005^3^

^1^Two GPs shared two lists at two separate surgeries, both in the intervention group. Weighted analysis taking this into account did not lead to significant change in the baseline characteristics

^2^Data are presented as mean ± SD or number (%)

^3^Significant at *p* < 0.01

^4^Significant at *p* < 0.001

### Interrater reliability

The interrater reliability was found to be Kappa = 0.93 (*p* < 0.0001), 95 % CI (0.73, 1). Additional analysis with the data divided into quintiles showed Kappa = 0.90 (*p* < 0.0001), 95 % CI (0.71, 1). Analysis for each rater pair separately yielded Kappa values ranging from 0.85 to 0.93 (further details available upon request).

### Primary outcome

**Fig. 2:**
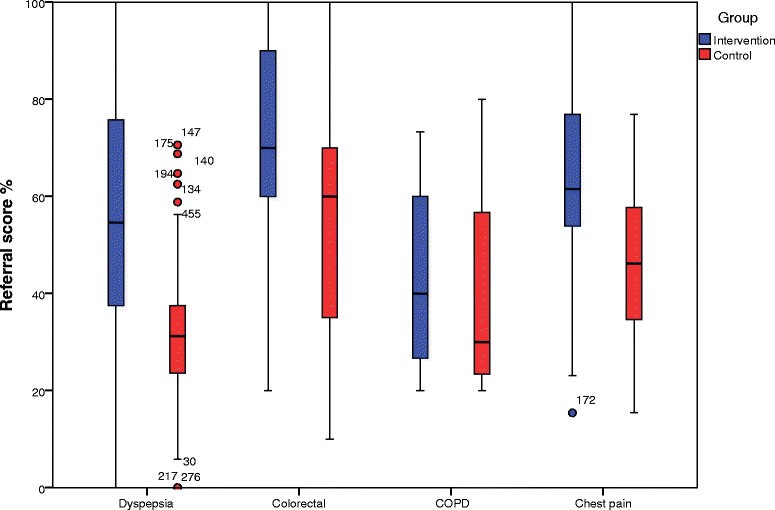
Referral quality. Referral quality by diagnostic group, uncorrected for clustering, presented as percentages

The average referral quality in each of the four diagnostic groups, not corrected for clustering, was higher in the intervention than the control group (Fig. 2). Large variations in quality were seen across all four diagnostic areas, both in the intervention and control groups. Table 4 presents these findings, not corrected for clustering, showing highly significant improvements in referral quality scores in all clinical areas except COPD. However, the absolute number of COPD referrals was very low.

**Table 4:** Average referral quality by diagnostic group, uncorrected for clustering^1^

	Intervention	Control	*p*-value
Dyspepsia	57.3 (53.0, 61.7)	31.4 (28.7, 34.1)	<0.001
Suspected colonic malignancy	70.1 (65.6, 74.6)	53.4 (48.5, 58.3)	<0.001
COPD	43.4 (7.1, 79.6)	40.0 (0, 83.3)	0.857
Chest pain	61.9 (56.2, 67.5)	45.3 (38.3, 52.3)	<0.001

^1^Presented as mean and 95 % CI

Baseline evaluation of the main outcome variable (referral quality) demonstrated that it was nearly normally distributed. Naïve analysis of data—that is, using a mixed models approach without adding the level of GP surgery into the analysis—was compared with a model including the GP surgery as clustering unit. Adding the random intercept to the model decreased the −2 log likelihood by 4529.25–4493.50 = 35.75. This is highly significant according to the Chi squared distribution with one degree of freedom. From the above analysis, the intraclass correlation coefficient (ICC) was calculated to be 0.14 (95 % CI (0.02, 0.25)). The final model corrected for whether the GP was a board certified specialist, centred mean GP patient list size, GP hospital experience (in years), and categorised GP surgery size. GP experience (in years) was removed from the model because it had no impact on the outcome of interest and had no clear association with referral quality. In addition, GP experience was clearly correlated with being a GP specialist, and its inclusion would thus reduce the power of the analysis without adding further insight.

Allowing the result to vary randomly at the level of the referring GP further decreased the −2 log likelihood of the baseline model to 4419.70 and reduced residual variance. The addition of a third level added complexity to the model and only changed the estimation of the intervention effect by 2 percentage points. It was therefore decided to keep the two level model proposed in the methods paper [22].

With this model, the multi-level regression analysis suggested a significant intervention effect with an approximately 20 % higher referral score in the intervention group. In the final model, adjustment reduced the effect estimate to 18 % (95 % CI (11, 25), *p* < 0.001) (Table 5). The model suggests that board certified GPs produced referrals that were closer to the referral template (9 %, 95 % CI (4, 14), *p* < 0.001), whereas longer hospital experience during a GP’s career predicted slightly less complete referrals (−2 %, 95 % CI (−3, −1) *p* < 0.001). Larger GP surgeries also tended to produce higher quality referrals, but this association was not statistically significant. A Q-Q normality plot of residuals from the model showed no violation of normality assumptions.

**Table 5:** Intervention effect estimates

	Regression coefficient	95 % CI	*p*-value
Crude^1^	20.25	10.23, 30.27	*p* < 0.001
Adjusted^2^	18.00	11.03, 24.98	*p* < 0.001
- GP specialist yes/no	9.19	4.39, 13.99	*p* < 0.001
- GP list size (centred)	0.02	−0.01, 0.05	*p* = 0.196
- GP experience in hospital (in years)	−2.06	−3.08, −1.05	*p* < 0.001
- GP surgery size^3^	4.81	−2.73, 12.35	*p* = 0.211

^1^Baseline model with intervention effect with random intercept

^2^Adjusted for the variables listed below

^3^Categorised into binary variable 0–3 GPs and 3–6 GPs

Because only roughly 50 % of the intervention group referrals were sent to the intervention hospital electronic address, we performed a non-protocol multilevel model analysis comparing the quality of referrals between the intervention GPs who used the referral address and those intervention GPs who did not. We found a referral quality difference that was approximately as large as between the intervention and control group in the main analysis (21.9 %, 95 % CI (16.5, 26.2), *p* < 0.001).

## Discussion

The current paper presents the impact of the dissemination of a referral template on referral quality. The intervention improved referral quality by 18 %, which is presumably clinically relevant. Our finding is consistent with reported increases in referral quality in similar intervention studies [41, 42], whereas another study reported a smaller effect size [43]. However, as discussed in a Cochrane review on the subject, there have been few studies aimed at improving the quality of referrals, and several have had methodological weaknesses [44].

The current study suggests that dissemination of referral templates combined with local follow-up measures can indeed improve referral quality in the communication between primary and specialist health care, which is consistent with the conclusions in the Cochrane report [44]. The data also suggest that being a board certified GP improves the quality of written communication to specialist health services as measured by the referral scoring system. Surprisingly, a GP’s experience as a hospital doctor does not appear to predict referrals that include more of the content requested by hospital consultants. Years of experience as a GP showed no association with the outcome of interest and was left out of the model entirely. This may suggest that it is the communication and collaboration between hospital-based specialists and dedicated GPs that can produce better referral quality, and not the experience of the GP or the presence of a referral template per se.

However, it is important to recognise that while a hospital physician will try to prioritise received referrals based on the risk of serious illness, many referrals are sent for reasons other than ruling out or diagnosing disease. These can include patient reassurance, reduction of medico-legal risk, handing over of care, or to obtain a second opinion [45]. Others have shown that factors including GP gender and GP speciality can affect referral rates and have discussed whether higher professional insecurity and/or higher responsiveness to patient demand may explain some of this variation [46]. It is conceivable that some of these factors also affect referral content, and that referrals are not purely based on the GP’s perception of the patient’s individual risk of serious disease.

As shown above, in this study the quality of the referrals varied between the four clinical areas, with referrals for suspected colonic malignancy scoring highest (average 70.1 % in the intervention group and 53.4 % in the control group) and referrals for patients with COPD scoring lowest (43.3 vs. 40.0 %). We have found no comparable studies in which referrals for different clinical areas have been scored using the same scoring technique, and it is therefore hard to assess whether this quality difference is a general phenomenon. However, the referral template for colonic malignancy contained the fewest requested clinical data points, and the scientific basis for these points was better documented than those for the COPD or dyspepsia referrals. This may suggest that for referral templates to be effective, simpler templates based on solid scientific research may be more acceptable and user-friendly in clinical practice than complicated templates.

The referral scores varied widely for each diagnostic area (Fig. 2). This confirms the pre-trial clinical suspicion of variation, which was one of the motivations for this study. Especially within the area of dyspepsia, wide variation was seen in both the intervention and control groups, with several outliers. This suggests that some GPs produce referrals of high quality, regardless of the referral intervention, and that some general practitioners in the intervention group took no interest in the intervention at all. This wide range in performance has also been noted when the referral rate has been assessed [46, 47]. Although uniformity does not necessarily equate to quality, it is intuitive that some degree of increased uniformity in referral quality would improve equity in the health care delivered to patients.

Adding interactions to the model showed a significant interaction between intervention status and being a board certified GP. This suggested a stronger intervention effect amongst those who were board certified. This was felt to be adequately represented in the model by the combination of the terms ‘board certified GP’ and ‘hospital experience’.

The weighted Kappa analysis equates to ‘almost perfect’ agreement among raters (Kappa 0.81–1.00) according to Landis and Koch [48]. Even considering the increasing Kappa values with increasing categories discussed above, this shows not only excellent overall reliability, but also excellent agreement between all three rater pairs.

This study has several limitations. The referral templates used in the project were developed according to international literature and local practices. Referrals were scored based on how closely they followed this referral template. Conscientious GPs were therefore likely to score very high on the referral score, and this could bias the results in favour of the intervention. Nonetheless, the scoring system does equate with referral quality measurement scores used in other referral evaluation studies [2, 5]. In addition, further work in this project aimes to assess whether the presence of a greater quantity of relevant clinical information improves the quality of the health care process, and consequently this scoring system seemed appropriate. It is possible that some of the effect size noted above was caused by GPs who took a special interest in the study.

A further weakness is that the current project does not provide a clear indication of the proportion of the referring GPs who actively used the referral intervention. The referral templates were distributed and follow-up visits were arranged to ensure adherence to the study protocol. As presented above, only about 50 % of the referrals from intervention GPs were sent to the newly formed intervention electronic address. This suggests a fairly modest uptake of the intervention, and the non-protocol analysis showed a higher intervention effect amongst those GPs who actively utilised the electronic address. Because intention-to-treat analysis was used, this has probably attenuated the intervention effect. Similar difficulties have been seen in other projects, with an uptake as low as 18 % in a referral intervention study for patients with lower bowel symptoms [49]. However, a recent Norwegian project using referral guidance as an electronic pop-up reported that the 88 % of the intervention GPs used the intervention ‘all the time’ [20].

Many known barriers to changes of behaviour and application of clinical knowledge exist, including lack of knowledge/awareness, lack of applicability to the individual patient and organisational factors [50]. Feedback from the GPs in this project suggests that the intervention was used and appreciated, but also easily forgotten in hectic everyday clinical work. If wider application of referral guidelines is to be considered, careful assessment should be undertaken to identify barriers to their use and to indicate tailored interventions to overcome these barriers, as this has been shown to be more likely to improve professional practice [51].

Another limiting factor is that the rate of inclusion of relevant patients is unknown. During the inclusion phase, regular reviews took place to assess the rate of inclusion at the hospital, which was estimated to be approximately 60 % of possible outpatient candidates. A completely accurate figure is not available, as this would require a manual search of the charts of every patient with an outpatient appointment, which is beyond the ethical approval of this project.

It is also clear that more patients were recruited from intervention GP surgeries than control GP surgeries. The total number of listed patients in the intervention and control group GP surgeries was very similar (19,347 vs. 19,906). The study did not have access to referral rates, and it is not clear whether these varied between the practices. There is no clear indication of major baseline differences between the GP surgeries and the study patients that can explain the difference in inclusion. One possible explanation is that the focus on the four diagnostic areas in the intervention offices caused more patients to be referred, but this cannot be demonstrated from the current data.

It is important to note that making referral information more in line with the hospital physicians’ wishes does not automatically predict improved outcomes. It is conceivable that referrals that are more pleasing to the hospital consultant may give a false sense of precision in the evaluation and prioritisation of referrals. For colorectal cancer, a review of symptoms and diagnostic tests in primary care suggests that few symptoms and signs are sensitive and specific enough to be used to identify patients at higher risk. However, it does indicate that referral guidelines and symptom combinations may aid in this process [52]. Further research is necessary to identify the clinical symptoms, signs or tests that will allow a clear prediction of risk in order to guide the information included in referrals. As we wait for this guidance, we must use the tools currently available, including good communication and clear and informative referrals.

Healthcare costs are rising [21]. For healthcare managers and policymakers, it would be helpful if the implementation of referral guidelines can improve patient prioritisation, as suggested in a recent Norwegian report supporting the use of referral guidelines and their implementation in the electronic health record [53]. As discussed, caution must be exercised, because a more precise referral may not predict better quality of care. Further analysis within this project is currently underway to determine whether improved referral quality results in a meaningful change in patient prioritisation and quality of care.

## Conclusion

This cluster randomised study assessing the impact of the dissemination of referral templates coupled with intermittent surgery visits by study personnel demonstrates a significant and substantial improvement in the measured quality of referrals in the intervention group. Further analysis is underway to determine whether this improvement in observed referral quality will predict an increase in the quality of care delivered to individual patients. For future studies, it appears prudent to utilise simple referral guidance, developed in collaboration between primary and secondary care. The referral guidance will need to be embedded in the patient record system to ensure its implementation.

## Additional file

Additional file 1:
**Referral templates.** (PDF 89 kb)
