# Supplementary material for: Impact of referral templates on the quality of referrals from primary to secondary care: a cluster randomised trial
Source: BMC Health Serv Res. 2015 Aug 29;15:353. doi: 10.1186/s12913-015-1017-7 (PMC4553012; doi:10.1186/s12913-015-1017-7)
Supplement: Additional file 1: — Referral templates. (PDF 89 kb) [file 12913_2015_1017_MOESM1_ESM.pdf]

Suspected colorectal cancer referral template

In addition to standard information in the referral we request more detailed information about the following:

- change in bowel habit
- blood in stool
- weight loss
- family history of colorectal cancer
- previous medical history of bowel disease or results of previous bowel investigations
- results from digital rectal examination
- iron deficiency anaemia
- clinical findings at abdominal examination
- results of faecal occult blood test
- the general practitioners clinical suspicion

Dyspepsia referral template

In addition to standard information in the referral we request more detailed information about the following:

- dysphagia
- odynophagia
- anorexia
- weight loss
- haematemesis
- malaena
- vomiting
- medications (especially NSAID, acetylsalicylic acid, bisphosphonates)
- nocturnal symptoms
- symptom duration
- previous peptic ulcer disease
- previous upper gastrointestinal tract operations
- jaundice
- cervical lymphadenopathy
- hepatomegaly
- anaemia
- if <50 years – HP (Helicobacter pylori) status

Chest pain referral template

In addition to standard information in the referral we request more detailed information about the following:

- family history (sudden death or premature coronary disease in first degree relatives)
- coronary risk factors (smoking, obesity (BMI), diabetes, hypertension, hypercholesterolaemia)
- comorbidity (peripheral vascular disease, cerebrovascular disease, kidney failure)
- chest pain (localisation, duration, radiation, effect of activity, effect of nitroglycerin)
- dyspnoe (precipitating cause, progression, dyspnoe at rest)
- syncope (if yes – details)
- palpitations
- progression of symptoms
- level of functioning (see Canadian Cardiovascular angina grading scale)
- general practitioners clinical assessment/suspicion
- clinical examination (BT/pulse, jugular venous stasis, auscultation of heart and lungs, peripheral oedema)
- ECG
- blood tests (including haemoglobin, creatinine, LDL cholesterol)

Canadian Cardiovascular Society angina grading scale	
I	Symptoms only during strenuous or prolonged physical activity
II	Symptoms only during physical activity
III	Symptoms with everyday physical/living activities
IV	Symptoms with minimal activity or symptoms at rest

COPD referral template

In addition to standard information in the referral we request more detailed information about the following:

- main symptoms (including duration, degree, dynamics)
- dyspnoea (at rest, at exertion etc)
- cough
- mucous production
- recurrent infections requiring treatment
- reaction to irritants (cold, change in weather etc.)
- level of functioning in everyday life
- haemoptysis
- weight loss (amount, duration)
- stable clinical situation, or acute exacerbation
- smoking status/previous smoking history
- treatment attempted, effect?
- comorbidities and updated list of medications (incl. inhaled medications)
- spirometry values
- chest X-ray description, if available
